# A Core Filamentation Response Network in *Candida albicans* Is Restricted to Eight Genes

**DOI:** 10.1371/journal.pone.0058613

**Published:** 2013-03-14

**Authors:** Ronny Martin, Daniela Albrecht-Eckardt, Sascha Brunke, Bernhard Hube, Kerstin Hünniger, Oliver Kurzai

**Affiliations:** 1 Septomics Research Center, Friedrich Schiller University and Leibniz Institute for Natural Product Research and Infection Biology – Hans Knoell Institute, Jena, Germany; 2 BioControl Jena GmbH, Jena, Germany; 3 Department of Microbial Pathogenicity Mechanisms, Leibniz Institute for Natural Product Research and Infection Biology- Hans Knoell Institute- and Friedrich Schiller University, Jena, Germany; 4 Center for Sepsis Control and Care, Jena University Hospital, Jena, Germany; 5 Friedrich Schiller University Jena, Jena, Germany; New Jersey Medical School, University of Medicine and Dentistry of New Jersey, United States of America

## Abstract

Although morphological plasticity is a central virulence trait of *Candida albicans*, the number of filament-associated genes and the interplay of mechanisms regulating their expression remain unknown. By correlation-based network modeling of the transcriptional response to different defined external stimuli for morphogenesis we identified a set of eight genes with highly correlated expression patterns, forming a core filamentation response. This group of genes included *ALS3*, *ECE1*, *HGT2*, *HWP1*, *IHD1* and *RBT1* which are known or supposed to encode for cell- wall associated proteins as well as the Rac1 guanine nucleotide exchange factor encoding gene *DCK1* and the unknown function open reading frame orf19.2457. The validity of network modeling was confirmed using a dataset of advanced complexity that describes the transcriptional response of *C. albicans* during epithelial invasion as well as comparing our results with other previously published transcriptome studies. Although the set of core filamentation response genes was quite small, several transcriptional regulators are involved in the control of their expression, depending on the environmental condition.

## Introduction

The formation of filaments by *Candida albicans* is an essential attribute of this species with direct implications for tissue invasion and virulence. An extensive list of *in vitro* conditions including pH, temperature, nutrient sources, CO_2_ concentration and serum has been described to favor growth of *C. albicans* in either yeast or filamentous morphotype [1], [2]. Whereas in early reports filamentous forms were described as “virulent” or “invasive” in contrast to the “commensal” yeast morphotype, numerous studies now suggest that morphological plasticity rather than a single morphotype are required during infection. This hypothesis is backed by the observation that mutants locked in either yeast (*efg1*Δ*/cph1*Δ) or filamentous (*tup1*Δ/*nrg1*Δ) morphotype show reduced virulence in infection models [3], [4]. The central role of the shift towards filamentous growth has been emphasized in studies using mutant strains that retained their switching ability but showed either extended filamentation after derepression of *UME6* from a tet-controlled promotor or induced reversion to yeast morphology due to tet-induced expression of *NRG1* [5], [6]. Filamentous growth may directly contribute to host damage by invasion of epithelial or dendritic cells [7], [8] and modulate the antifungal immune response [9], [10]. In addition, among the several genes that have been identified to be expressed in a morphotype dependent manner, “filament-specific” genes and their products have frequently been characterized as virulence factors. The most prominent examples for this include the adhesin and iron-recruitment protein Als3 [11], [12] and the adhesin Hwp1 [13]. However, many other genes described as “filament-specific” have only been studied under selected conditions and the transcriptomic changes associated with filamentation of *C. albicans* independent of the external stimulus have not been fully elucidated.

Two major signal cascades, the cAMP pathway and the MAP kinase cascade and their terminal transcription factors Efg1 and Cph1 control the formation of filaments [14], [15], [16]. Depending on the external stimulus, other pathways like the pH response cascade may contribute to filamentation, but Efg1 often remains the terminal transcription factor of these pathways [16], [17]. Besides activation, derepression is also crucial for the induction of filamentous growth [18]. The major repressor complex consists of the regulator Tup1 and its DNA-binding partner Nrg1 and prevents expression of hyphae-associated genes [4], [19], [20]. Detachment of this complex from target gene promoters is controlled by remodeling of chromatin structures and contributes to filamentous growth [21], [22]. Overexpression or constitutive expression of *NRG1* prevents filamentation [20], [23] and deletion of both or either *TUP1* or *NRG1* results in a hyperfilamentous phenotype ([4], [19], [20]. Within this study we used three different, well- defined stimuli to induce hyphal growth of *C. albicans*. A combination of transcriptome analyses and network modeling helped to define a core filamentation response for this fungal pathogen. Only eight genes were part of a group of genes with highly correlated expression pattern which was up- regulated in hyphae independent from the environmental condition. Analysis of expression patterns in *C. albicans* regulatory mutants confirmed that regulation of this core filamentation response is complex and depends on the environmental stimulus.

## Results

### Filamentous Growth Dynamics in *Candida albicans*

**Figure 1:**
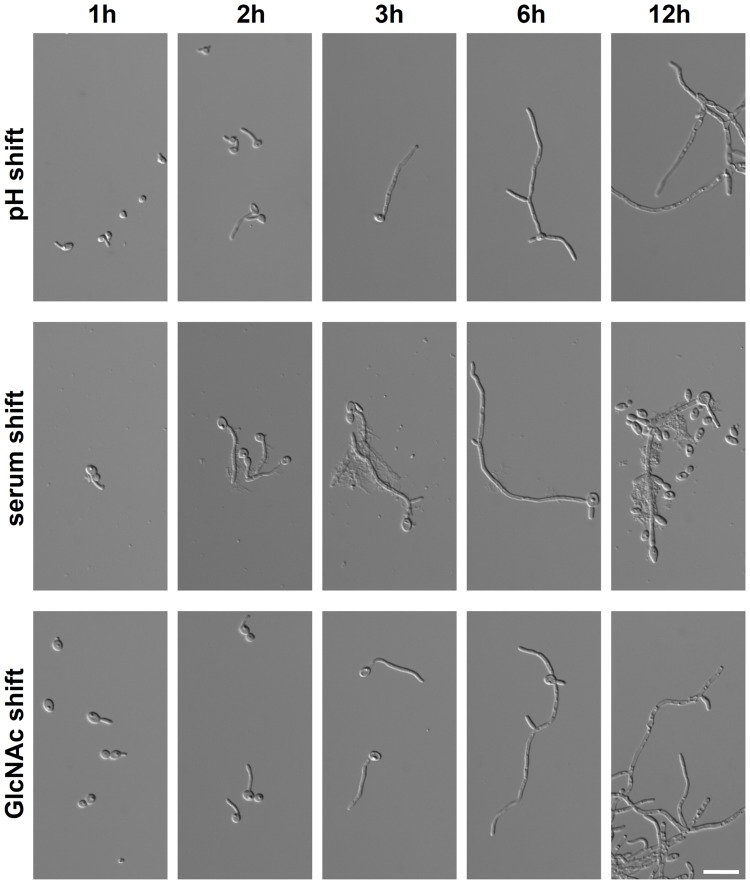
Dynamics of filamentous growth in *Candida albicans*. Cells of *C. albicans* SC5314 strain were incubated under filament- inducing conditions in time course experiments for up to 12 h. For pH shift, cells were transferred from a M199 pH4 preculture to M199 pH8. For the other shifts, cells were transferred from a preculture in SDG medium into either SDG with 10% human serum (serum shift) or into SDN medium with 20 g/l N- acetylglucosamine as exclusive carbon source (GlcNAc shift). Note, that precultures were already grown at 37°C over night, so there was no additionally temperature shift effect to stimulate germ tube formation. For later time points, individual hyphae are shown instead of hyphal conglomerates but were identical in average length. Precipitates visible in serum induction regulary occur with human serum, baccterial contaminations were excluded. Scalebar: 20 µm.

Three well- defined stimuli were used to induce filamentation in stationary phase *C. albicans* yeast cells: (i) a shift from pH4 to pH8, (ii) the addition of 10% human AB serum to the medium and (iii) the change of the carbon source from 2% glucose to N- acetylglucosamine (for details see Material and Methods section). Human serum was used to be as close as possible to the natural environment in the human host. In time course experiments, we observed two stages of filamentation for all three shifts, germ tube formation during the first two hours after stimulation followed by hyphal elongation and branching (Figure 1). For transcriptional profiling, filament- inducing conditions were optimized to ensure a maximum comparability of germ tube formation kinetics.

**Figure 2:**
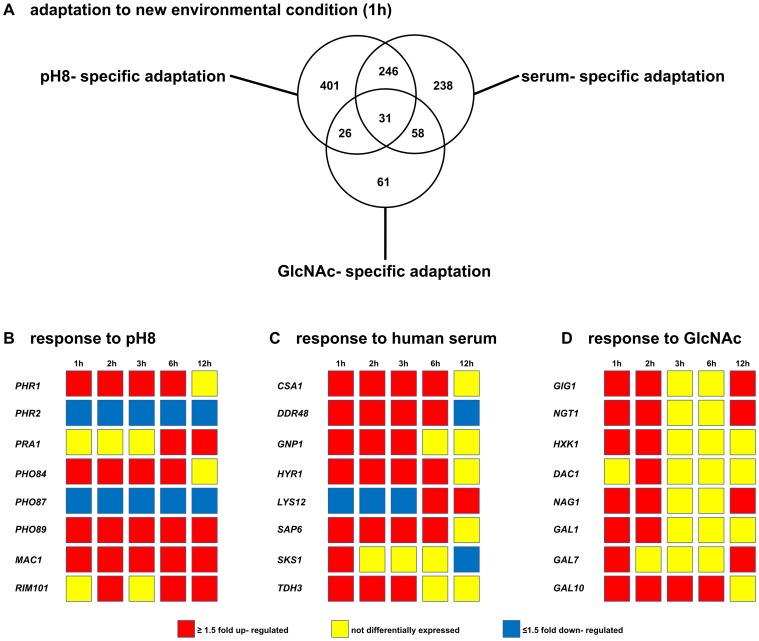
Shift- specific gene expression patterns in *Candida albicans* hyphae. A summary of the data from the whole genome DNA microarrays used for this study. Genes showing fold changes of at least 1.5 were evaluated for significance (p≤0.05) and illustrated in blue for down- regulation and red for up- regulation. Genes were marked in yellow as not differentially expressed. (A). Differentially expressed genes for all three shifts at 1 h. (B–D) The expression dynamics of genes closely linked to the pH shift(B), the serum shift (C) or the change of the carbon source from glucose to N- acetylglucosamine (D) are shown. The presented data were taken from the whole genome DNA microarrays used for this study. The fold changes of at least 1.5 were evaluated for significance (p≤0.05) and illustrated in blue for down- regulation and red for up- regulation. Genes were marked in yellow as not differentially expressed.

For each condition, 1×10^6^ cells/ml from the overnight preculture were transferred into prewarmed medium (at 37°C) which either promoted yeast or hyphal growth. By this, we avoided a temporary shift from 37°C to lower temperatures and back which might delay hyphal development. Using a serum concentration of 10% resulted in kinetics which were most closely related to the pH and GlcNAc shift. For each time point, cells were checked for germ tube formation (Figure 1, 1 h and 2 h) or hyphae with visible septa and/or branches (Figure 1, 3–12 h). Control cultures were checked before RNA isolation for stable yeast morphology.

**Figure 3:**
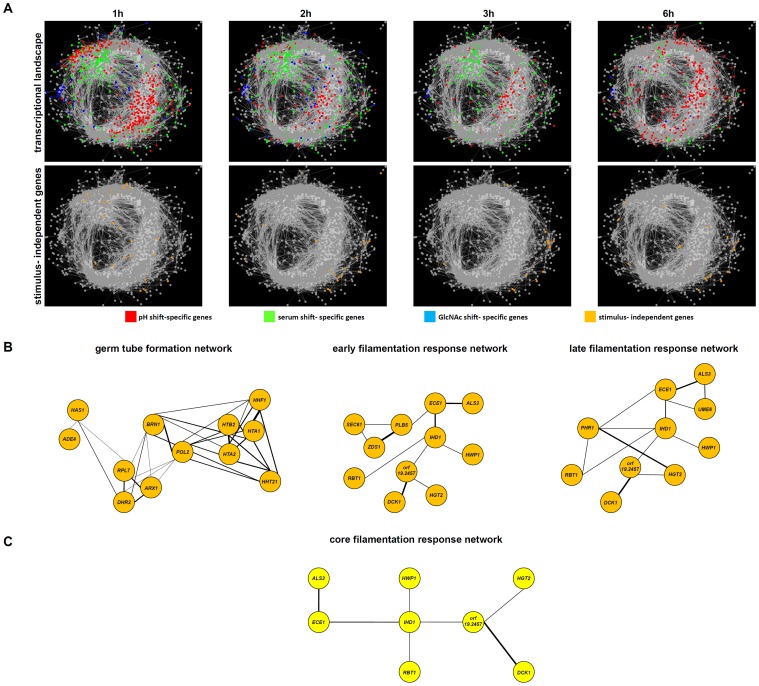
Transcriptional landscape modeling of *Candida albicans* hyphae. (A) Based on data from whole genome DNA microarrays, correlation- based networks of gene expression were modeled using the Cytoscape software visualizing the overall transcriptomic response to the three stimuli in the time-course of filamentation. For germ tube formation, only genes which were differentially expressed during 1 h and/or 2 h in all three shifts were used for modeling and only those which were linked directly to each other with high correlation (≥0,75) were integrated into the networks, leading to the identification of an upregulated set of early filamentation genes (early filamentation network EFN, B) and a group of genes downregulated during induction of filamentation (germ-tube formation network GFN, B). The same modeling was performed for hyphal elongation with genes which were differentially expressed at 3 h and 6 h in all three shifts, resulting in the description of a single network of upregulated genes (late filamentation network, LFN, B). (C) Integration of the time-point networks defined the core filamentation response consisting of a network of eight genes showing highly correlated expression patterns.

Serum-induced hyphae reverted to budding filaments and even yeast cells again, whereas reversions were barely detected throughout the observation periods when using the other two protocols (Figure 1, 12 h).

Total RNA was isolated for each of the standardized conditions at five distinct time points (1 h, 2 h, 3 h, 6 h and 12 h) in three independent biological replicates and used for transcriptome analysis. Raw data are available at ArrayExpress (http://www.ebi.ac.uk/arrayexpress/, accession number E-MEXP-3675).

### Shift- associated Gene Expression Patterns

To confirm that the three experimental shifts induced specific transcriptional adaptation, overall transcription patterns were analyzed. A total of 704 genes changed their expression in response to pH shift from pH4 to pH8 at the 1 h time point. More than 50% (401 out of 704, Figure 2 A and Table S1) were specific for the pH shift, while 303 genes were also differentially expressed in either the serum or the GlcNAc or both (Figure 2 A). As expected we detected the down- regulation of *PHR2*, which is known to be expressed at acidic pH [24] and the simultanous up- regulation of *PHR1* [25] (Figure 2 B). In addition, the alkaline induced gene *PRA1* showed an increased expression at 6 h and 12 h (Figure 2 B). In response to the serum shift, 573 genes showed either an up- or a down- regulation at the 1 h time point. Less than half of them (238) were specific for serum- induced germ tubes (Figure 2 A and Table S1). *CSA1*, *DDR48*, *SAP6* and *HYR1* were highly up- regulated in serum- stimulated hyphae as reported previously [26], [27]. Consistent with reversion to yeast morphology they were no longer expressed or even down- regulated after the hyphae- to yeast reversion at 12 h (Figure 2 C). In total, only 176 genes were differentially expressed 1 h after the carbon source change (Table S1) with 61 genes specific for the GlcNAc shift. Among those were genes associated with uptake and processing of GlcNAc, including *NGT1 (*encoding a GlcNac specific transporter), *HXK1 (*encoding a GlcNAc- kinase), *NAG1* (encoding a Glucosamine-6-phosphate deaminase) and *DAC1* (encoding a N-acetylglucosamine-6-phosphate deacetylase), which have previously shown to reflect adaptation to GlcNAc [28], [29], [30] (Figure 2 D). In addition, genes *GIG1*, *GAL1*, *GAL7* and *GAL10* were also up- regulated during the GlcNAc shift as shown previously [30], [31], [32] (Figure 2 D).

Taken together, these analyses clearly show, that each of the three shift induces a highly specific transcriptional adaptation which is predicted to involve several different signaling cascades based on prior knowledge [16].

### Transcriptional Landscape Modeling Reveals a Core Filamentation Response Network

For further analyses, we focused on genes which were differentially expressed for all three protocols. For this term, we only took the first 6 hours into account as at the 12 h time point reversion of serum- induced hyphae to yeast cells was observed (Figure 1). For all differentially regulated transcripts we calculated pairwise Pearson correlation coefficients and their significance over all 12 test conditions of the experiment using R software 2.14.1. For visualization, we selected all pairs of transcripts with correlation coefficients larger than 0.75 (all showing a p-value smaller than 0.0013). Visualization was done with Cytoscape 2.8.1 [33] using the edge-weighted spring embedded layout. Based on gene expression correlation, we identified two different networks during the event of germ tube formation. These networks comprise all transcripts that are differentially regulated at 1 h and/or 2 h by all three stimuli. Only genes which were directly linked with each other are shown in the networks. The first network, consisting of down- regulated genes, contains genes that are related to either ribosome function, RNA metabolism or chromatin remodeling and histones (germ-tube formation network [GFN]; Figure 3 B). The central hubs of this network were the *C. albicans BRN1* homolog, which encodes a putative condensin I and *POL2*, encoding DNA polymerase epsilon (Figure 3 B) Interestingly, this network was linked to the first hour of stimulation only, as most of these genes were no longer differentially expressed at later time points. However, some of them were up- regulated at the 12 h time point (Table S1). The second network was characterized by up- regulated genes which mainly have already been linked to filamentation such as *ALS3*, *ECE1*, *RBT1* and *IHD1* (early filamentation network [EFN], Figure 3 B). Germ tube formation was followed by hyphal elongation, characterized by longer and branching hyphae (Figure 1). In contrast to the earlier stage of hyphal growth, only a single network of up- regulated genes was identified at this stage, which was highly related to the EFN (late filamentation network [LFN], Figure 3 B). The LFN consisted of known hyphae- associated genes including *ALS3*, *ECE1*, *HWP1* and *IHD1* as well as of the regulatory gene *UME6*, which was shown to be crucial for hyphal elongation (Figure 3 B) [34]. *PHR1*, whose up- regulation is a typical transcriptional response to alkaline pH (Figure 2 B), was up- regulated in elongated hyphae in all three shifts and was therefore included within the network, sugesting a role which is independent from pH sensing (Figure 3 B). It should be noted that the networks are based on correlation in expression profiles. Therefore, some genes which were differentially expressed in all three stimuli were not showing up as they were not linked to the nodes of the aforementioned networks. The two most prominent examples are *EED1*, a regulator of hyphal elongation [35] which was only up- regulated at 6 h (Table S1) and the repressor gene *NRG1*, the only gene which was down- regulated during hyphal elongation phase in all three shifts (Table S1, 3 h and 6 h). Interestingly, this down- regulation disappeared in serum- stimulated cells during the process of hyphae- to yeast- reversion while it was still down- regulated in pH- and GlcNAc- stimulated hyphae which did not undergo reversion (Table S1, 12 h). A combination of the EFN and the LFN resulted in a minimal set of eight genes which were part of the early filamentation network as well as of the late filamentation network: *ALS3*, *DCK1*, *ECE1*, *HGT2*, *IHD1*, *HWP1*, *RBT1*, orf19.2457. All of them could be linked to each other into a network, which was defined as the core filamentation response network (CFR) of *C. albicans* (Figure 3 C). This group of genes was up- regulated in germ tubes and hyphae, independently from the growth phase as well as the stimulating environmental condition.

### *IHD1* and orf19.2457 are not Essential for Hyphal Development

**Figure 4:**
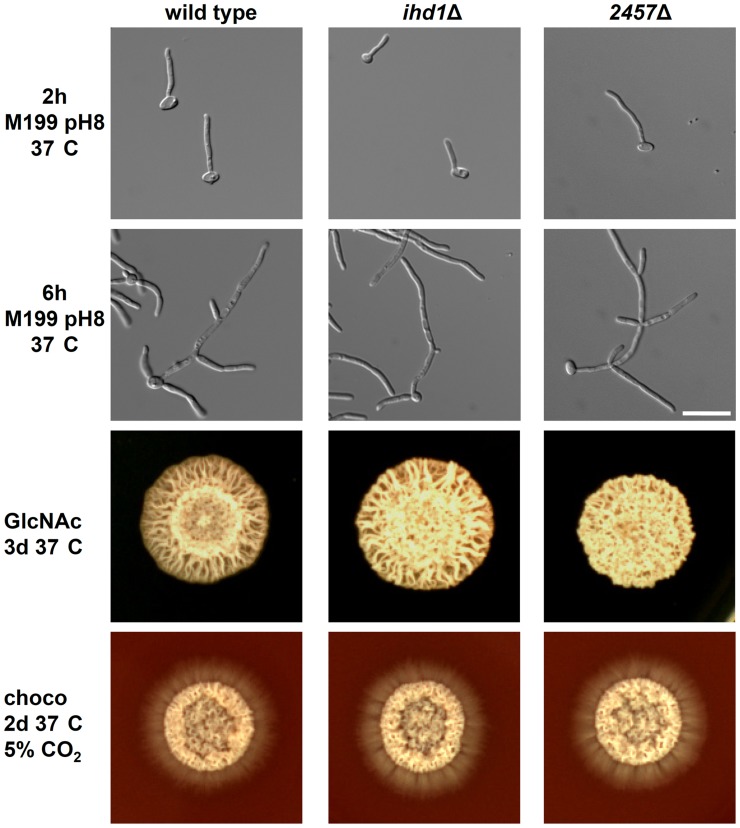
Deletion of *IHD1* and orf19.2457 did not affect filamentous growth. Cells of the C. albicans wild type strain SC5314 and mutants *ihd1*Δ and orf19.2457Δ were transferred from an overnight preculture in M199 pH4 grown at 37°C to M199 pH8 and incubated for 2 h or 6 h prior to microscopy. For hyphal growth on solid media in either plates with 20 g/l N-acetylglucosamine as sole carbon source or chocolate agar, 1×10^6^ cells/ml from a preculture grown in SDG minimal medium at 37°C overnight, were dropped onto plates and incubated for 2 or 3 days prior to photography. Scalebar: 20 µm.

The genes *IHD1* and orf19.2457 were central hubs of the core filamentation response network (Figure 3 C). Both not yet characterized ORFs were deleted in a PCR- based gene targeting approach and the resulting homozygous mutants were tested for their ability to develop hyphae or not. Neither the deletion of *IHD1* nor orf19.2457 did affect the ability to form hyphae under the tested conditions (Figure 4), indicating none of them is actually required for the yeast to hyphae transition. These findings fit to the fact that previously described mutants lacking CFR genes *ALS3* [36], *ECE1* [37] and *RBT1* [38]were still able to form filaments. In contrast, mutants lacking *HWP1* [39] and *DCK1* [40], [41] displayed defects in hyphal development. No information is available for the effects of *HGT2* deletion on filamentation.

### Comparison with the Results from Other Transcriptome Studies

To validate our findings, we analyzed the expression of the identified CFR genes in published transcriptome analyzes dealing with filamentation of *C. albicans* in similar or more complex experimental settings such as the interaction of fungal and human host cells. For that purpose studies using whole genome *C. albicans* expression arrays were identified from PubMed focusing on two groups of datasets: (i) whole- genome approaches dealing with hyphal induction with single, well- defined stimuli such as serum or cAMP, (ii) transcriptome analyses dealing with more complex conditions during host- pathogen interaction with a clearly defined yeast morphology as starting point and filamentation occurring within the analyzed time-frame. In addition, two recent studies using RNAseq [42] or tiling arrays [43] were included into the analysis.

**Figure 5:**
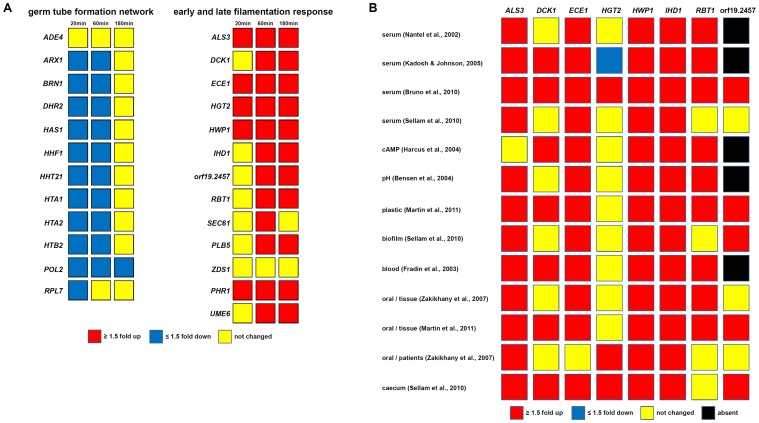
Expression pattern of the core filamentation response genes in selected transcriptome studies. Transcriptional data from selected studies were analyzed for information about the eight core filamentation response network genes. As different technologies and normalization pattern were used, it is only possible to provide information about up (red)- or down- regulation (blue) or a no change of expression (yellow) of the indicated genes. The open reading frame orf19.2457 was partially not part of the microarray design in some studies and were marked absent (black). (A) Expression dynamics for the genes of the early and late filamentation networks as well as of the germ tube formation network during the invasion of human oral epithelial cells (TR146 cell line) with *C. albicans* wild type SC5314. The dataset were taken from the study of Wächtler and coworkers [44]. (B) Expression dynamics for the core filamentation response genes in different transcriptome studies covering more hyphae- inducing conditions thant those which were used in this study.

Wächtler *et al.* analyzed the transcriptional dynamics of *C. albicans* during invasion of human oral epithelial cells using the same array as in our analyses [44]. In this study, very early time points of the invasion process (20 and 60 minutes) as well as later time point (180 minutes) were examined. The CFR as defined by our analyses was up- regulated for all time points (Figure 5 A). With only one exception (*ADE4*), genes of the GFN showed a decreased expression during the early time points 20 and 60 minutes, but were no longer differentially expressed at the later 180 time point (Figure 5 A). In addition, all genes of the EFN and LFN except *ZDS1* showed strong up-regulation at 60 min *post infection* (Figure 5 A).

Within the core filamentation response genes, *ALS3*, *ECE1*, *HWP1*, *IHD1* and *RBT1* were mostly found to be up- regulated during the switch from yeast to hyphae in experiments including serum shift, pH shift, cAMP shift, induction via plastic surfaces, biofilms or fungal interaction with oral epithelial cells, blood or caecum cells (Figure 5 B). Fitting best to our findings were the results from a RNA- seq based transcriptional profile of serum- induced hyphae [42] were all aforementioned CFR genes were up- regulated (Figure 4 B). As *ALS3*, *ECE1*, *HWP1* and *RBT1* were long time predicted hyphae- specific genes [16], it was of interest to find out the dynamics of the four additional CFR genes. *IHD1* was up- regulated in all analyzed transcriptome studies including an *in vivo* study with clinical samples from patients with oral candidosis (Figure 5 B) [45]. *DCK1* was up- regulated in studies analyzing filaments induced by serum [18], [42], cAMP [46], contact with plastic surfaces or human oral epithelial cells [35] caecum cells and human blood [43], [47], although other studies failed to detect a differential expression of this gene (Figure 5 B). *DCK1* was clearly up- regulated in our study of alkaline- induced hyphae, but not in a previous one [48] (Figure 5 B).In contrast to the other CFR genes, *HGT2* was only found to up- regulated in one serum study [42] and caecum cells [43], but interestingly also in patients suffering from oral candidosis [45]. In another study, however, *HGT2* was down- regulated in serum- induced hyphae [18] (Figure 5 B).

### Regulation of CFR Genes

**Figure 6:**
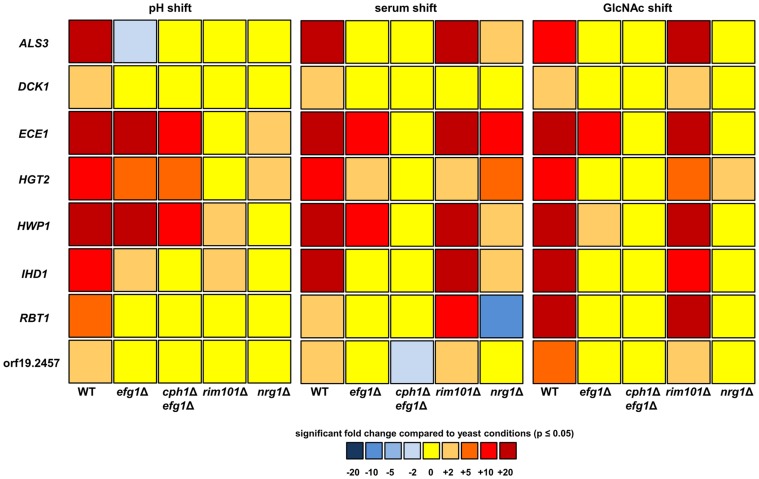
Regulation of core filamentation response gene expression. Mutants lacking the transcriptional regulators Efg1, Cph1, Rim101 and Nrg1 were grown for 3 h at 37°C (pH shift: from pH4 to pH8, serum shift: addition of 10% human serum, GlcNAc shift: N- acetylglucosamine as carbon source). Expression of the eight core filamentation response genes was analyzed by quantitative RT PCR in three independent experiments and gene expression was normalized against the housekeeping gene *ACT1* (actin) and a common reference (wild type, 6 h in YPD at 37°C). To calculate the fold change of expression, relative gene expression of hyphae- inducing conditions (e.g. pH8) were compared to yeast promoting conditions (e.g. pH4). The illustrated fold changes were evaluated for significance (p≤0.05, student’s t test).

Except for *DCK1*, all of the CFR genes are characterized by large 5′ intergenic regions before the open reading frames with sizes between 2 kb and 6 kb (Table 1). On average, 5′ intergenic regions including promoters in *C. albicans* are 500 to 1000 bp [49]. Argimon et al. have previously suggested that 5′ intergenic regions of hyphae- specific genes are unusually long compared to *C. albicans* genes in general [50]. All of the CFR promoters contained putative binding motifs for transcription factor Efg1 and with the only exception orf19.2457 they also contained Nrg1 response elements (Table 1). Based on these *in silico* observations, the expression of the CFR genes was analyzed in non- filamentous mutants lacking *EFG1* (*efg1*Δ and *cph*1Δ/*efg1*Δ) and the hyperfilamentous *nrg1*Δ mutant. Additionally, we tested the *rim101*Δ mutant, which formed wild type- like filaments during the serum and GlcNAc shift, but not after the change from pH4 to pH8. An increased expression of the CFR genes was observed in the wild type after 3 h hyphal growth in all three shifts (Figure 6). Interestingly, *ECE1*, *HWP1*, *HGT2* and *IHD1* were strongly expressed in *efg1*Δ and partially in *cph*1Δ/*efg1*Δ but not in *rim101*Δ during the pH shift, suggesting that Rim101 is more important for the regulation of these genes under pH inducing conditions than Efg1 (Figure 6). For the two other protocols, there was no induction of CFR genes in the *cph*1Δ/*efg1*Δ double mutant. The results suggest an impact of Cph1 on the regulation of both *ECE1* and *HWP1*, as there was still an increase of expression of these genes in *efg1*Δ, but not in *cph*1Δ/*efg1*Δ (Figure 6). In the filamentous *nrg1*Δ mutant, the basic level of CFR gene expression was higher than in wild type and therefore the increase of expression was often not significant. However, some genes were still strongly induced in this mutant in response to filament inducing conditions, such as *ECE1* during serum shift, indicating that a combination of derepression and activation contributes to the high levels of expression (Figure 6).

**Table 1:** *In silico* promoter analysis of core filamentation response genes.

CFR gene	size of intergenic region	binding motifs for Efg1 E box elements:C A N N T G	Binding motifs for Nrg1 Nrg1 response elements: (A/C) (A/C/G) C C C T
***ALS3***	2963 bp	1× CAACTG (−11)	1× AACCCT (−327)
		2× CAAATG (−1403, −2479)	1× CCCCCT (−78)
		3× CAATTG (−946, −1161, −1278)	
		1× CATTTG (−118)	
***DCK1***	1383 bp	2× CAGTTG (−486, −500)	1× AACCCT (−376)
		1× CAAATG (−1026)	1× CCCCCT (−467)
		2× CATTTG (−871, −976)	
***ECE1***	3197 bp	1× CAAGTG (−1586)	1× AACCCT (−284)
		1× CAACTG (−2138)	1× CGCCCT (−2149)
		2× CAAATG (−1048, −1098)	
		3× CAATTG (−199, −759, −1604)	
		2× CATTTG (−493, −710)	
***HGT2***	6399 bp	1× CAAGTG (−1124)2× CATGTG (−754, −5238)3× CAACTG (−3546, −4461, −5572)3× CAAATG (−674, −4884, −5747)3× CAATTG (−2371, −3957, −5154)5× CATTTG(−60, −1436, −3097, −4006, −5143)	1× AACCCT (−4825)1× CCCCCT (−67)
		2× CATGTG (−754, −5238)	1× CCCCCT (−67)
		3× CAACTG (−3546, −4461, −5572)	
		3× CAAATG (−674, −4884, −5747)	
		3× CAATTG (−2371, −3957, −5154)	
		5× CATTTG (−60, −1436, −3097, −4006, −5143)	
***HWP1***	2080 bp	1× CAACTG (−586)	2× AACCCT (−344, −1280)
		1× CAATTG (−1141)	1× CACCCT (−781)
		1× CATTTG (−1926)	
***IHD1***	2367 bp	1× CAACTG (−1726)	1× ACCCCT (−514)
		2× CAATTG (−643, −1409)	
		2× CATTTG (−688, −1913)	
***RBT1***	3414 bp	1× CAGTTG (−2930)	1× AACCCT (−2307)
		2× CAACTG (−1324, −2100)	
		2× CAAATG (−2051, −2611)	
		3× CAATTG (−1198, −2873, −3301)	
		2× CATTTG (−1599, −3112)	
**orf19.2457**	3348 bp	2× CAAGTG (−3188, −3218)	no binding motif found
		1× CAACTG (−131)	
		3× CAAATG (−1039, −2328, −3145)	
		2× CAATTG (−1323, −2671)	

in brackets: starting nucleotide of motif within the intergenic region.

## Discussion

Many different stimuli can induce filamentous growth of *C. albicans* and a lot of protocolls were used to trigger the morphological change from unicellular yeasts into germ tubes and later hyphae [16]. For this study, we have chosen three well- defined stimuli which require change of only one condition compared to yeast-promoting media. In addition, all three shifts could be performed at a temperature of 37°C which reflects temperature in the natural human habitat of *C. albicans*. Dilution of stationary phase culture has previously been described to result in the formation of germ-tubes due to the release of quorum-sensing induced inhibition of filamentation [51]. However, in our experiments, control conditions favoring yeast growth were diluted in an identical way as experimental conditions and no induction of filamentation was observed. In contrast to the aforementioned study [51] we have not used YPD with a neutral pH as preculture and medium for yeast- like growth which might explain the absence of a quorum- sensing effect. In concordance with a trigger-specific induction of filamentation. *C. albicans* responded differently to each of the three shifts leading to gene expression pattern which were characteristic for each shift (Figure 2). Of the three shifts we used, human serum was the one where hyphae to yeast reversion occurred at the earliest time point (between 6 and 12 h), whereas this process was barely detected for the other two shifts in the 12 h time course (Figure 1).

A combination of classical transcriptome analysis tools with network modeling led to the identification of a surprisingly small core filamentation response (CFR) in *C. albicans* which is differentially regulated during the formation of hyphae independent of the external stimulus. The algorithms employed for network modeling have originally been developed to analyze and visualize large datasets for protein-protein or protein-DNA interaction together with mRNA quantification [52] In the correlation- based networks used for analysis of large-scale transcriptome data in this study, the algorithm allows a clustering of co-regulated genes and – in the setting of different input conditions leading to an identical response (in this case filamentation) – the definition of core gene sets that are directly related to the response and independent of the input condition. Using this approach, only eight genes fulfilled the criteria for a core filamentation response: *ALS3*, *DCK1*, *ECE1*, *HGT2*, *HWP1*, *IHD1*, *RBT1* and orf19.2457. These genes constitute a core filamentation network of co-regulated genes. *ALS3*, *ECE1* and *HWP1* have long been considered prototypic hyphal associated genes and their identification confirms the validity of our approach [16]. All of those encode for cell-wall/cell-membrane proteins, which is also true for *DCK1*, *HGT2*, *IHD1* and *RBT1* according to published data or sequence based predictions. Only the protein encoded by orf19.2457 may have no surface localization based on *in silico* analyses and nothing is known about its function.

Hardly any information is available for *HGT2*, a gene homologous to the high-affinity glucose transporter *HGT1*. However, the gene is located next to a cluster containing also the galactose metabolism genes *GAL10*, *GAL7* and *GAL1* [53], although it is separated from the other three genes by a very large intergenic region [54]. These genes share similar or equal motifs in their promotor regions suggesting a regulatory function of the transcription factor Cph1 [53]. However, the expression dynamics were quite different. While the *GAL* genes were very specific for the GlcNAc shift (Figure 2 D, Table S1), *HGT2* was up- regulated in all three shifts for the majority of time points (Table S1). Therefore, its expression seems to be separately regulated from the *GAL* genes and is at least under pH8 inducing conditions independent from Cph1 (Figure 6).

The central hub of the core filamentation response network is the gene *IHD1*. The function of the encoded protein is unknown so far, although it might be GPI- anchored and therefore should localize on the fungal surface. Previously, *IHD1* has been described as a hyphal induced for serum and temperature triggered filamentation [55]. Rbt1 is an adhesin related to Hwp1 and has been involved in mating and biofilm formation [56]. Dck1, a guanine nucleotide exchange factor for the Rac1 GTPase, is dispensable for serum-induced filamentation but required for matrix-embedded induced filamentous growth, a condition that was not tested in our study [41]. Our data indicate that Dck1-Rac1 may have a stimulus independent function in filamentation. As *RAC1* itself was not differentially expressed, this might suggest a Rac1- independent function of Dck1. Based on our data, *DCK1* and *HWP1* are the only genes in the core filamention response that are necessary for filamentation at least in certain conditions [39], [41] while all other CFR network genes are effectors of a filamentation rather than being required for this morphological process. This is also reflected by the fact that well known regulators of filamentation including *EFG1*, *HGC1*, *UME6*, *CPH1* and many others are not part of the CFR.

According to several transcriptome studies from the past decade and to the list of hyphae- induced genes which was published by Sudbery [16], genes like *DDR48*, *HYR1*, *SAP4-6* and *SOD5* might have been expected to be part of the core filamentation response. However, none of these genes was included due to the fact that they were up- regulated in one or two filament- inducing shifts but not simultaneously in all three shifts. All of them were up- regulated during the serum shift and partially the pH (*DDR48*, *SOD5*) or the GlcNAc shift (*HYR1* and *SAP6*). *DDR48*, *HYR1* and *SOD5* were however not differentially expressed or even down- regulated after serum- induced hyphae reverted to yeasts at the 12 h time point (Table S1). Taken together, these four genes are associated to the filamentation response in a broader meaning, but they are not as stimulus-independent as the aforementioned core filamentation response genes.

Validation of the CFR network with data from other experimental settings confirms that it defines a minimum number of genes which can be used to assess morphogenesis in most if not all experimental settings. This is of potential interest for several applications: Using transcriptional quantification of these genes, newly generated mutants defective in morphogenesis can easily be tested for an expression pattern that resembles yeast or hyphal morphology, aiding in the identification of mutants displaying a dissociation of phenotype from transcriptional pattern as described for the *C. albicans* Δ*hgc1* mutant [57]. Similarily, quantification of genes regulated during early morphogenesis could assist in further elucidating the role of filamentation in interaction of *C. albicans* with immune cells by providing a tool for early detection of a switch towards filamentation in the transcriptional program.

Despite the low number of CFR genes, the regulation of these genes is apparently complex. Quantification of CFR expression in several regulator mutants suggested that CFR expression is governed by overlapping and at least partially redundant molecular mechanisms. In addition, whereas regulation of the CFR genes occurs independent of the external stimulus for filamentation, the regulatory mechanisms leading to induction differ between the stimuli, suggesting that stimulus-dependent contributions of several regulators rather than a universal master regulator govern CFR expression. A prominent example for this is the fact, that Rim101 is absolutely required for induction of all CFR genes in pH-shift induced filamentation, whereas it is dispensable in the other conditions.

Furthermore, the identification of the GFN which is specifically downregulated during the very early phase of hyphal development is consistent with the involvement of chromatin modification in the regulatory processes. Modification of histone proteins has shown to be involved in regulation of morphogenesis [58] and it was recently shown that chromatin remodeling plays an important role in allowing Nrg1 to get access to its target DNA sequences [21], [22].

In summary, we could show that correlation-based modeling of transcriptional analyses has proven useful to identify a core set of marker-genes characteristic for the important biological process of filamentation in *C. albicans*. Therefore, correlation- based network modeling might be suitable tool for further analyses of other processes in *C. albicans*.

## Materials and Methods

### Strains, Media and Growth Conditions

All *C. albicans* strains used in this study are listed in Table 2. Strains were routinely grown at 37°C in either M199 medium (9.8 g/l M199 powder, PAA; 35.7 g/l HEPES, 2.2 g/l sodium carbonate; adjusted to different pH values with either sodium hydroxide or hydrochloride acid) with pH4 or SDG medium (6.7 g/l YNB without amino acids, DIFCO, 20 g/l glucose). For hyphal induction, 1×10^6^ cells/ml were transferred from a stationary phase culture in M199 pH4 to M199pH8 (“pH shift”) or from a stationary phase culture in SDG to SDG with 10% human serum (PAA, “serum shift”) or SDN (6.7 g/l YNB without amino acids, DIFCO, 20 g/l N- acetlyglucosamine, “GlcNAc shift”). Cells were then incubated at 37°C for a total of 12 h.

**Table 2:** *C. albicans* strains used in this study.

strain name	Genotype	source
SC5314	wild type	[64]
SN87	*leu2Δ/leu2Δ, his1Δ/his1Δ, URA3/ura3Δ::imm434, IRO1/iro1Δ::imm434*	[59]
*tup1*Δ	*tup1::hisG/tup1::hisG, RPS10/rps10::*CIp10*-URA3*	[35]
*nrg1*Δ	*nrg1::hisG/nrg1::hisG*, *RPS10/rps10::*CIp10-URA3	[35]
*efg1*Δ	*efg1::hisG/efg1::hisG-URA3-hisG*	[3]
*cph1*Δ/*efg1*Δ	*cph1::hisG/cph1::hisG/efg::hisG/efg1::hisG-URA3-hisG*	[3]
*rim101*Δ	*rim101::hisG/rim101::hisG-URA3-hisG*	[65]
*IHD1/ihd1*	SN87, *IHD1/ihd1::CmLEU2*	this work
*ihd1*Δ	SN87, *ihd1::CmLEU2/ihd1::CdHIS1*	this work
*2457/2457*Δ	SN87, *19.2457/19.2457::CmLEU2*	this work
*2457*Δ	SN87, *19.2457::CmLEU2/19.2457::CdHIS1*	this work

### Construction of *C. albicans* Mutants

The genes *GIT2*, *IHD1*, orf19.1344 and orf19.2457 were deleted in the background strain SN87 [59] which is auxotrophic for histidine and leucine. Gene deletions were performed with a PCR- based strategy using the plasmids pFA-CdHIS1 and pFA-CmLEU2 [60]. Transformations of *C. albicans* strains were performed with the established lithium- acetate method [61].

### Microscopy

Routinely, cells were analyzed by DIC microscopy, which was performed on a Zeiss AxioObserver Z.1 (Carl Zeiss, Göttingen and Jena, Germany).

### Transcriptome Analysis

Total fungal RNA was isolated by a hot phenol- chloroform method previously described in more detail [35]. Quality and quantity of the RNA were checked by Agilent Bioanalyzer 2100 (Agilent Technologies). For whole genome expression studies, sample RNA was labeled with Cy5-CTP and hybridized with a Cy3- labeled common reference RNA on *C. albicans* DNA microarrays (ClinEuroDiag, Brussels, Belgium). Slides were hybridized, washed and scanned with a Genepix 4000B (Molecular Devices) as described previously [62]. After quality control, arrays were preprocessed using R software version 2.14.1 (http://www.r-project.org). Printtiploess and Gquantile methods were used for normalization. A linear model was fit to the normalized data. Transcripts were regarded as being significantly differentially expressed when they showed an absolute fold change of larger than 1.5 and an FDR adjusted t-test p-value of less than 0.05. Raw data of the microarrays are available at ArrayExpress (http://www.ebi.ac.uk/arrayexpress/) with the accession number E-MEXP-3675 for wild type arrays.

### Network Modeling

For all significant differentially regulated transcripts we calculated pairwise Pearson correlation coefficients and their significance over all 15 test conditions of the experiment using R software 2.14.1. For visualization, we selected all pairs of transcripts with correlation coefficients larger than 0.75 (all showing a p-value smaller than 0.0013). Visualization was done with Cytoscape 2.8.1 [33] using the edge-weighted spring embedded layout.

### Determination of Gene Expression Levels

Quantitative RT PCR was performed using the Brilliant III Ultra Fast SYBR Green qRT PCR Kit (Agilent Technologies) on a Stratagene Mx3005P (Agilent Technologies) with 100 ng/µl RNA as template. To determine the fold changes of gene expression we used the ΔΔCt method [63]. Gene expression was normalized against *ACT1* as housekeeping gene and the aforementioned common reference RNA as control.

## Supporting Information

Table S1 Differentially expressed genes during filamentous growth induced by different stimuli. All genes listed in the tables were differentially expressed (≥1.5 fold with p≤0.05) in at least one condition at one time point. If a gene was differentially expressed in more than one condition, it is listed in the conditions column with "HS_SDG & pH8–pH4". HS_SDG means that the gene is differentially expressed in serum- treated cells (HS for human serum) compared to control cells grown in SDG medium. SDN_SDG means that the gene is differentially expressed in GlcNAc- treated cells (SDN for medium with GlcNAc as carbon source) compared to control cells grown in SDG. pH8_pH4 means that the gene is differentially expressed in cells grown in M199 medium with pH8 compared to those grown in M199 medium with pH4.
(XLS)
